# Two-pore Channels (TPC2s) and Nicotinic Acid Adenine Dinucleotide Phosphate (NAADP) at Lysosomal-Sarcoplasmic Reticular Junctions Contribute to Acute and Chronic β-Adrenoceptor Signaling in the Heart*

**DOI:** 10.1074/jbc.M115.684076

**Published:** 2015-10-05

**Authors:** Rebecca A. Capel, Emma L. Bolton, Wee K. Lin, Daniel Aston, Yanwen Wang, Wei Liu, Xin Wang, Rebecca-Ann B. Burton, Duncan Bloor-Young, Kai-Ting Shade, Margarida Ruas, John Parrington, Grant C. Churchill, Ming Lei, Antony Galione, Derek A. Terrar

**Affiliations:** From the ‡ Department of Pharmacology, BHF Centre of Research Excellence, University of Oxford, Mansfield Road, Oxford OX1 3QT,; the § Faculty of Life Science, University of Manchester, Manchester M13 9NT, and; the ¶ Department of Physiology, Anatomy and Genetics, Sherrington Building, University of Oxford, Sherrington Road, Oxford OX1 3PT, United Kingdom

**Keywords:** adrenergic receptor, Ca2+/calmodulin-dependent protein kinase II (CaMKII), calcium imaging, calcium intracellular release, cardiac hypertrophy, endoplasmic reticulum (ER), heart, lysosome, nicotinic acid adenine dinucleotide phosphate (NAADP)

## Abstract

Ca^2+^-permeable type 2 two-pore channels (TPC2) are lysosomal proteins required for nicotinic acid adenine dinucleotide phosphate (NAADP)-evoked Ca^2+^ release in many diverse cell types. Here, we investigate the importance of TPC2 proteins for the physiology and pathophysiology of the heart. NAADP-AM failed to enhance Ca^2+^ responses in cardiac myocytes from *Tpcn2*^−/−^ mice, unlike myocytes from wild-type (WT) mice. Ca^2+^/calmodulin-dependent protein kinase II inhibitors suppressed actions of NAADP in myocytes. Ca^2+^ transients and contractions accompanying action potentials were increased by isoproterenol in myocytes from WT mice, but these effects of β-adrenoreceptor stimulation were reduced in myocytes from *Tpcn2*^−/−^ mice. Increases in amplitude of L-type Ca^2+^ currents evoked by isoproterenol remained unchanged in myocytes from *Tpcn2*^−/−^ mice showing no loss of β-adrenoceptors or coupling mechanisms. Whole hearts from *Tpcn2*^−/−^ mice also showed reduced inotropic effects of isoproterenol and a reduced tendency for arrhythmias following acute β-adrenoreceptor stimulation. Hearts from *Tpcn2*^−/−^ mice chronically exposed to isoproterenol showed less cardiac hypertrophy and increased threshold for arrhythmogenesis compared with WT controls. Electron microscopy showed that lysosomes form close contacts with the sarcoplasmic reticulum (separation ∼25 nm). We propose that Ca^2+^-signaling nanodomains between lysosomes and sarcoplasmic reticulum dependent on NAADP and TPC2 comprise an important element in β-adrenoreceptor signal transduction in cardiac myocytes. In summary, our observations define a role for NAADP and TPC2 at lysosomal/sarcoplasmic reticulum junctions as unexpected but major contributors in the acute actions of β-adrenergic signaling in the heart and also in stress pathways linking chronic stimulation of β-adrenoceptors to hypertrophy and associated arrhythmias.

## Teaser

**Background:** The Ca^2+^-releasing messenger nicotinic acid adenine dinucleotide phosphate (NAADP) acts via lysosomal two-pore channels (TPC2).

**Results:** *Tpcn2*^−/−^ cardiac myocytes showed reduced acute responses to β-adrenoreceptor stimulation and chronically reduced cardiac hypertrophy and arrhythmogenesis.

**Conclusion:** Acute and chronic effects of cardiac β-adrenoreceptor stimulation depend on NAADP acting via TPC2 in lysosomes.

**Significance:** NAADP/TPC2 signaling pathways offer new strategies for cardiac therapeutics.

## Introduction

NAADP^11^ is the most potent Ca^2+^-mobilizing messenger yet described, and it plays important roles in a wide variety of animal and plant cells (1, 2). NAADP uniquely evokes Ca^2+^ release from lysosomes (3–8), an action demonstrated to require two-pore channels (TPCs) (9–12). TPCs are evolutionarily ancient members of the superfamily of voltage-gated ion channels found in both plant and animal cells and localize to the endo-lysosomal system. TPC2 proteins are exclusively expressed on vacuolar or lysosomal membranes (13). Consisting of 12 transmembrane domains, they have so far been shown to be regulated by NAADP, inositol lipids, and voltage (13). The formation of functional channels seems likely to require dimerization and allows for physiological expression of homo- or heterodimers (14, 15), whereas responses to NAADP likely depend on the association of an unidentified NAADP-binding protein (16, 17). In this complex manner, TPCs are able to act as a signaling hub to regulate endo-lysosomal ion homeostasis with NAADP responsible for lysosomal contributions to Ca^2+^-signaling events.

The diversity of cellular mechanisms that involve NAADP signaling is remarkably broad, including egg fertilization, insulin secretion, and neuronal differentiation (18). Recent work on the Ebola virus has added another example of the potential importance of this pathway, showing that inhibition of NAADP-mediated Ca^2+^ signaling drastically reduces infectivity (19). The use of *Tpcn* knock-out mouse lines demonstrates the roles for TPC proteins in physiological processes, both at the cellular and whole-organism levels (9, 11, 19–30).

We have previously observed that β-adrenoreceptor stimulation can increase NAADP levels in cardiac muscle (31, 32), and functional evidence supports a link between β-adrenoreceptor stimulation and the effects of NAADP (31, 33). Recent work is also suggestive of a role for NAADP in arrhythmias induced by acute β-adrenoreceptor stimulation (34). However, the role of TPCs in cardiac tissue and the contribution of this pathway to the pathology surrounding chronic β-adrenergic stimulation have yet to be described.

There has been some discussion over which cations pass through TPCs in lysosomes and in particular whether these channels are permeable to Ca^2+^ (28, 35–37). A recent study shows that TPCs are a requirement for NAADP-mediated lysosomal Ca^2+^ release (11) and that the actions of NAADP on lysosomes involve permeation of TPC channels by Ca^2+^ (11, 38).

Two isoforms of TPCs are expressed in human and mouse cells, and we have investigated NAADP-evoked Ca^2+^ release and β-adrenoreceptor signaling in murine cells and mice that have been genetically modified to lack TPC2 protein (*Tpcn2*^−/−^). This isoform was targeted because it has been shown to be specifically expressed in lysosomal and late endosomal membranes (9).

Our observations are the first to demonstrate the importance of TPC2 proteins for both acute and chronic consequences of stimulation of β-adrenoceptors in heart muscle, and they provide yet another major role for these ion channels, the NAADP pathway and acidic organelles, in the (patho-) physiology of Ca^2+^ signaling.

## Experimental Procedures

### 

#### 

##### Cell Isolation, Guinea Pig

Male guinea pigs (300–500 g) were killed in accordance with Schedule 1 of The Animals (Scientific Procedures) Act 1986 (HMSO). The heart was rapidly excised and washed in a modified Tyrode's solution containing EGTA and heparin to prevent clots from forming in the small coronary circulation. The heart was then mounted on a constant pressure Langendorff perfusion system for retrograde perfusion via the aorta. Following a 2-min initial perfusion with a modified Tyrode's solution (in mm: NaCl 136, KCl 5.4, NaHCO_3_ 12, Na^+^ pyruvate 1, NaH_2_PO_4_ 1, MgCl_2_ 1, EGTA 0.04, glucose 5; gassed with 95% O_2_, 5% CO_2_ to maintain a pH of 7.4), digestion was carried out with a Tyrode's solution (50 ml) of identical composition, but which lacked EGTA and contained 24–28 mg of collagenase type II (Worthington) and 0.1 mm CaCl_2_. Following digestion, the left atrium was separated and placed in high K^+^ storage medium (in mm: KCl 70, MgCl_2_ 5, K^+^ glutamine 5, taurine 20, EGTA 0.04, succinic acid 5, KH_2_PO_4_ 20, HEPES 5, glucose 10; pH 7.2 with KOH). Single cells were isolated by gentle trituration and stored at 4 °C in high K^+^ storage medium until use. Ventricular tissue was dissected into several pieces and underwent mechanical agitation for a further 5 min in collagenase solution. Isolated myocytes were filtered through a 250-μm mesh, and collagenase was replaced with Dulbecco's modified Eagle's medium (DMEM, Invitrogen) warmed to 37 °C. Ventricular myocytes were stored in DMEM at room temperature and used within 9 h of isolation.

##### Creation of TPC2^−/−^ Mice

Mice lacking the TPC2 protein were created as described previously (9, 11).

##### Cell Isolation, Mouse

Mice (13–18 weeks old) were killed in accordance with Schedule 1 of The Animals (Scientific Procedures) Act 1986 (HMSO). Hearts were excised, tied to a cannula via the aorta and perfused through a syringe containing a pre-gassed (with 95% O_2_, 5% CO_2_) solution containing (mm) NaCl 125, NaHCO_3_ 25, KCl 5.4, NaH_2_PO_4_ 1.2, MgCl_2_ 1, CaCl_2_ 1.0, glucose 5.5, pH 7.4, together with heparin (10 units/ml) and streptokinase (100 units/ml). The cannula was then removed from the syringe and mounted onto a Langendorff perfusion system. Once mounted, the hearts were perfused with a solution (36 °C) containing (mm) NaCl 130, KCl 5.4, MgCl_2_ 3.5, glucose 10, HEPES 5, NaH_2_PO_4_ 0.4; pH 7.4, and gassed with 95% O_2_, 5% CO_2_. After 3 min, this isolation solution was replaced with a further 50 ml of a similar solution that also contained 0.3 mg/ml collagenase (type II, Worthington) and 0.1 mm CaCl_2_. Following enzymatic digestion (for a maximum time of 7 min), the heart was removed from the cannula. The ventricles were cut into pieces, and the myocytes were released during several gentle suspensions, then filtered through 250-μm mesh, and stored at room temperature in medium containing (in mm) NaCl 130, KCl 5.4, MgCl_2_ 3.5, CaCl_2_ 0.1glucose 10, HEPES 5, NaH_2_PO_4_ 0.4, taurine 20; 0.1% bovine serum albumin; pH 7.4, and gassed with 95% O_2_, 5% CO_2_.

##### Electrophysiology

Cardiac myocytes were mounted in a perfusion bath placed at the stage of an inverted microscope and perfused with a physiological salt solution (PSS) containing (in mm) NaCl 125, NaHCO_3_ 25, KCl 5.4, NaH_2_PO_4_ 1.2, MgCl_2_ 1, glucose 5.5, CaCl_2_ 1.8 and oxygenated with 95% O_2_, 5% CO_2_ to maintain a pH of 7.4. The PSS used for mouse experiments was identical except that CaCl_2_ concentration was reduced to 1 mm. All electrophysiology was performed at 36 °C. Glass microelectrodes were manufactured from thin-walled, filamented borosilicate glass capillary tubing (GC150TF, Harvard Apparatus Ltd., Kent, UK). Electrode resistances of 3.0 ± 1.5 megohms were used for whole-cell experiments. During whole-cell recordings, a whole-cell patch solution was used containing(in mm) K^+^ aspartate 110, KCl 10, NaCl 5, MgCl_2_ 5.2, HEPES 5, K_2_ATP 5, pH 7.2, with KOH, to which Ca^2+^ indicator dye, caged NAADP, and inhibitors were added at the stated concentrations. Perforated patch was carried out using whole-cell patch solution plus amphotericin B at 250 μg/ml. Current clamp recordings were made using an AxoClamp 2A amplifier system (Axon Instruments). Cells were stimulated to fire action potentials using a 2-ms current pulse applied via the microelectrode at a rate of 1 Hz. Voltage clamp recordings were made using an AxoPatch 200B amplifier system (Axon Instruments). To stimulate the L-type Ca^2+^ current (*I*_CaL_), cells were maintained at a holding potential of −40 mV and stimulated by a 200-ms step depolarization to 0 mV at a rate of 0.2 Hz. *I*_CaL_ amplitude was measured as maximum current minus current at the end of the voltage pulse.

##### Ca^2+^ Fluorescence, Dye Loading, and Cell Stimulation Protocol

During whole-cell fluorescence experiments, Fluo-5F salt (100 μm) was added to the whole-cell patch solution. In mouse experiments, myocytes were incubated with Fluo-5F AM (20 μm) for 20 min. Ca^2+^ transients were stimulated at 1 Hz either by a patch electrode (all guinea pig experiments) or by carbon fiber electrodes placed at the side of the superfusion bath (mouse experiments).

##### Epifluorescence

Guinea pig atrial myocytes were visualized using a Leica DMIRB inverted microscope. Excitation light was provided by 470 nm LED illumination, passed through a 475 ± 15 nm bandpass interference filter, and transmitted through the objective. Emitted light was passed through a 520-nm long pass filter and collected using a photomultiplier system (Cairn Research Ltd., Kent, UK). Photomultiplier signals were directed through an amplifier, followed by a low pass 100 Hz electronic filter, digitized (Axon Digidata 1200, Axon Instruments), and recorded with pClamp software at an acquisition rate of 10 kHz.

##### Spinning Disk Confocal Microscopy

Guinea pig and mouse ventricular myocytes were visualized using a Nikon Axiovert 200 inverted microscope with attached Nipkow spinning disk confocal unit (CSU-10, Andor Technology, UK). Excitation light was provided by a 488-nm diode laser (Cairn Research Ltd.) passed though the Nipkow unit and delivered to the sample through the objective. Emitted light passed back through the CSU-10 unit and was detected using an Andor iXON897 EM-CCD camera (Andor Technology) at a frame rate of 50 Hz. Images were recorded and analyzed using Andor iQ software (Andor Technology).

##### Line Scan Confocal Microscopy

Mouse ventricular myocytes were imaged with a confocal microscope system that consisted of a Leica TCS NT scanning head coupled to a Leica DMIRB inverted microscope with a 100× oil immersion objective lens (1.2 NA, Leica) in line scan mode (2.6 ms per line). Excitation light (488 nm) was provided by an air-cooled 488-nm argon ion laser system (Uniphase Ltd.), and emitted light was collected at wavelengths above 515 nm using a long- pass filter. Images were recorded using Leica TCS NT software and analyzed using ImageJ software.

##### Analysis of Ca^2+^ Transient Data

For analysis, background fluorescence was subtracted, and multiple transients were averaged to collect data at a given time point. Data are presented as *F*/*F*0 such that fluorescence data are presented relative to diastolic fluorescence. In the case of experiments in which NAADP was photoreleased in guinea pig ventricular myocytes, there was a small baseline change, and data are presented as *F/F*0i where *F*0i is equal to diastolic fluorescence before photorelease of NAADP.

##### Electron Microscopy

Rabbit ventricular tissue was fixed in Karnovsky fixative (39), resin-embedded, and sectioned at 80 nm (Reichart Ultracut). These were post-stained with 2% uranyl acetate and Reynolds lead citrate. Images were obtained using transmission electron microscopy (Jeol 1200EX II).

##### Contraction Studies, Mouse Ventricular Myocytes

Cells were stimulated to contract via a patch electrode (permeabilized configuration), and contractile properties were studied using the IonOptix system (IonOptix Corp.) to measure sarcomere length. Cells were visualized via a ×40 oil objective using an IonOptix MyoCam (IonOptix Corp.) that sampled images at a frequency of 240 Hz. Sinusoidal optical density traces arising from the alternating light and dark bands of the contractile machinery were then transformed into a signal of sarcomere length by application of a fast Fourier transform by the IonWizard sarcomere length acquisition software (IonOptix Corp.). Length measurements were calibrated using a stage micrometer with 2-μm graduations such that the number of pixels/μm recorded by the MyoCam could be entered into the software as a fixed value. Amplitude of sarcomere shortening was calculated by deduction of systolic from diastolic sarcomere length and expressed as a percentage of resting sarcomere length. Analysis was performed using IonWizard 5 software (IonOptix Corp.). All values represent an average of 10 contractions.

##### Langendorff-perfused Whole Mouse Hearts

Mice were killed in accordance with Schedule 1 of The Animals (Scientific Procedures) Act 1986 (HMSO). Hearts were excised, cannulated via the aorta under a microscope, and perfused for 30 s with PSS solution containing streptokinase (100 units/ml) via a syringe connected to the cannula. After initial perfusion, the syringe was removed and the cannula mounted on a Langendorff apparatus for retrograde perfusion with PSS containing 1.8 mm CaCl_2_ (36 °C, gassed with 95% O_2_, 5% CO_2_) at a constant flow rate (3.5 ml/min). Hearts were allowed to beat spontaneously, and contractile force was measured by means of a hook placed through the ventricles near the apex, attached to a tension transducer. Resting tension was set between 0.5 and 1.0 g initially. Heart rate was calculated from the force signal as the reciprocal of the time interval between contractions. Inflow pressure was measured using a pressure transducer positioned close to the aorta so that changes in coronary artery resistance could be measured as pressure changes at a constant perfusion flow rate. Data were acquired and analyzed using Chart software (ADInstruments, UK), with a data acquisition sampling rate of 100 samples/s. The tension signal was low pass-filtered with a cutoff frequency of 50 Hz.

##### Photorelease of Caged NAADP

NPE-caged NAADP, synthesized in-house by a method described previously (40), was included in whole-cell patch solution for use in both atrial and ventricular myocytes at the concentrations stated. NPE-caged NAADP is inactive until “uncaged” by exposure to UV light. UV photolysis was provided by rapid (∼1 ms) arc-lamp flash directed through a 355-nm filter and transmitted to the cell via the objective (atrial cells, Cairn Photolysis System, Cairn Research Ltd.; ventricular cells, Rapp Photolysis System, Rapp OptoElectronic, Vedel, Germany).

##### Cell Superfusion with NAADP-AM

NAADP-AM was synthesized in-house by a method described previously (41). Loading of NAADP into the cytosol was achieved by rapid switching the extracellular solution to one containing the membrane-permeant acetoxymethyl ester of NAADP (allowing rapid access of NAADP-AM to the cytosol and subsequent liberation of NAADP following action of intracellular esterases).

##### Programmed Electrical Stimulation (PES)

To assess propensity to arrhythmias, Langendorff-perfused hearts from WT and *Tpcn2*^−/−^ mice were subjected to PES. Monophasic action potentials were recorded from the left ventricular epicardial surface.

Hearts from mice not previously exposed to chronic β-adrenergic stimulation were subjected to PES in the presence of 50 nm isoproterenol. Ventricular pacing first occurred with a progressively increasing pacing current (from pacing capture current to 35 mA). At each current amplitude, three bursts of 50 stimuli were delivered. The cycle length between each stimulus was held at 20 ms, and the bursts were separated by a 2-s pacing-free interval. Data were expressed as a cumulative percentage of pacing-free time during which ventricular arrhythmias were observed. A second set of experiments was then performed in which the cycle length was progressively reduced (from 90 to 10 ms), and the pacing current was held at 10 mA. These data were analyzed using the same method.

Hearts from mice treated for 2 weeks with isoproterenol were subjected to a similar protocol in the absence of further β-adrenergic stimulation. A pacing train of eight stimuli (S1) was delivered with a cycle length of 100 ms, with a single (S2) premature extra stimulus introduced at progressively shorter intervals until arrhythmia was induced or the ventricular refractory period was reached. For the burst pacing protocol, ventricular pacing was carried out with a train of 50 S1 at a cycle length of 20 ms. Pacing current amplitude was progressively increased from the threshold of ventricular capture until ventricular tachycardia or fibrillation was induced or a current of 35 mA was reached. Ventricular tachycardia was defined as six or more consecutive premature ventricular waveforms (tachycardia with regular waveforms defined as ventricular tachycardia, whereas ventricular fibrillation was characterized by irregular fibrillating waveforms).

##### Hypertrophy Studies

Cardiac hypertrophy was induced by administration of isoproterenol (ISO, Sigma) at 10 mg/kg/day for 14 days via osmotic mini-pumps (Alzet) implanted subcutaneously in 8–10-week-old WT and *Tpcn2*^−/−^ mice.

##### Echocardiography

Mice were anesthetized with Avertin (Sigma, 200 mg/kg) via intraperitoneal injection. Transthoracic M-mode echocardiographic recordings were performed using an Acuson Sequoia C256 system (Siemens) following a protocol described previously (42). Measurements taken at end-systole and end-diastole were averaged to calculate parameters of end-diastolic left ventricular posterior wall thickness, left ventricular mass, and fractional shortening.

##### Electrocardiography

To monitor cardiac rhythms, we carried out *in vivo* electrocardiographic analysis on mice anesthetized with isoflurane (2.5%). RR interval, P wave duration, PR interval, QRS, JT, and QT durations were recorded.

##### Statistics

Statistical comparisons were made using paired or unpaired Student's *t* tests or one- or two-way analysis of variance (with repeated measures if appropriate) followed by either Tukey, Bonferroni, or Dunnett's post hoc test. Where a data set could not be deemed normally distributed, a Mann-Whitney test was used instead. A statistically significant difference was concluded when *p* was < 0.05. All data are expressed as mean values ± S.E.

## Results

**FIGURE 1.**
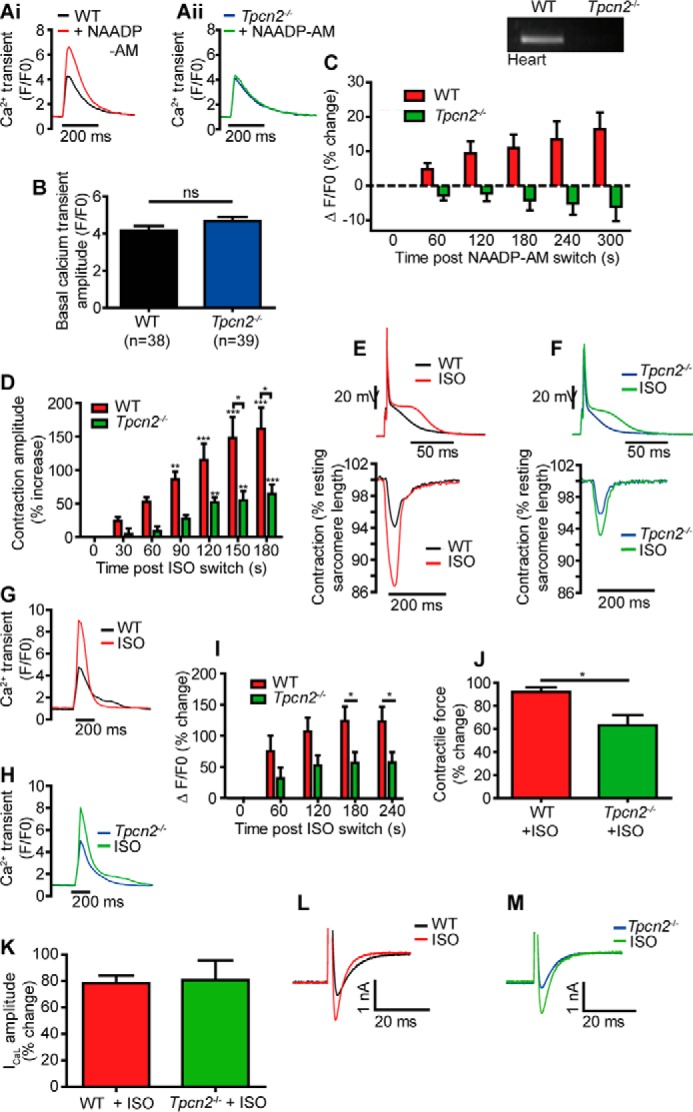
**NAADP actions were absent in cardiac ventricular myocytes from mice lacking TPC2 protein (*Tpcn2*^−/−^), and effects of β-adrenoreceptor stimulation on contractions and Ca^2+^ transients evoked by electrical stimulation were reduced in myocytes from *Tpcn2*^−/−^ as compared with wild-type myocytes.** *A, panels i* and *ii* show superimposed Ca^2+^ transients (Fluo-5F as probe, 1 Hz electrical stimulation) in myocytes before and after application of NAADP-AM (240 nm). *B* shows that the mean amplitudes of Ca^2+^ transients in the absence of drugs were similar in myocytes from *Tpcn2*^−/−^ and WT mice. *ns*, not significant. *C* shows mean data for effects of NAADP-AM (*n* = 20, both data sets). The *inset* to this panel shows that *Tpcn2* mRNA is found in hearts from WT but not *Tpcn2*^−/−^ mice. *D* shows mean increases in contraction amplitude during ISO (3 nm) application in myocytes from *Tpcn2*^−/−^ (*n* = 7) or wild-type mice (WT, *n* = 13, 1 Hz stimulation). *, *p* < 0.05; **, *p* < 0.01; ***, *p* < 0.001. *E* and *F* show representative action potential and contraction traces before and after ISO application in WT and *Tpcn2*^−/−^ cells. *G* and *H* show superimposed Ca^2+^ transients before and during ISO application in myocytes from *Tpcn2*^−/−^ or WT mice. Mean data are summarized in *I* (*n* = 15 for both data sets). *J* shows mean data from whole hearts perfused by the Langendorff technique. All observed effects of ISO were reduced in *Tpcn2*^−/−^ (*n* = 7) as compared with WT (*n* = 5); * indicates *p* < 0.05. *K* shows *bar graph* representing mean effects of isoproterenol on the amplitudes of L-type Ca^2+^ currents in response to step depolarizations from −40 to 0 mV (*red,* WT; *green, Tpcn2*^−/−^, *n* = 7 for both groups). Superimposed representative traces in the presence and absence of isoproterenol are shown in *L* (WT, *black* before and *red* after isoproterenol) and *M* (*Tpcn2*^−/−^, *blue* before and *green* after isoproterenol). The effects of isoproterenol were similar in WT and *Tpcn2*^−/−^.

We first demonstrated the absence of TPC2 expression at the mRNA level in cardiac tissue from *Tpcn2*^−/−^ mice, as shown in the inset to Fig. 1*C* (compared with wild type (WT)). Effects of exogenous NAADP were investigated in cells from these *Tpcn2*^−/−^ mice by superfusing myocytes with NAADP-AM, a membrane-permeant acetoxymethyl ester of NAADP (31, 33, 41). Myocytes were stimulated at 1 Hz to evoke Ca^2+^ transients accompanying action potentials. Application of NAADP-AM (240 nm) caused a significant increase in the amplitude of these Ca^2+^ transients (Fig. 1, *A, panel i,* and *C*, 16 ± 5%, *n* = 20) in myocytes from WT mice, but there was a striking failure of NAADP-AM to increase Ca^2+^ transient amplitude in ventricular myocytes from *Tpcn2*^−/−^ mice (Fig. 1, *A, panel ii,* and *C*, −6 ± 4%, *n* = 20). No difference was seen in Ca^2+^ transient amplitude between the two genotypes under control conditions (Fig. 1*B*). Therefore, the expression of TPC2 proteins is required for NAADP-mediated enhancement of Ca^2+^ transients in cardiac myocytes.

We then tested for the involvement of TPC2 in the response to β-adrenoreceptor stimulation. Fig. 1, *D–F,* shows effects of isoproterenol (3 nm) on contraction of myocytes electrically stimulated at 1 Hz. Increases in contraction amplitude caused by β-adrenoreceptor stimulation were greatly reduced in myocytes from *Tpcn2*^−/−^ mice (64 ± 14%; *n* = 7) as compared with the increases observed in WT myocytes (161 ± 32%; *n* = 13, *p* < 0.05). Action potential recordings made during the contraction study showed no difference between WT and *Tpcn2*^−/−^ cells under control conditions but showed a smaller increase in the “late plateau” phase of *Tpcn2*^−/−^ action potentials during exposure to ISO (an increase in amplitude of 8.3 ± 1.6 mV in myocytes from *Tpcn2*^−/−^ mice, *n* = 7, as compared with 13.0 ± 1.5 mV in myocytes from WT mice, *n* = 13, *p* < 0.05).

In view of the evidence presented above, and given that the late plateau phase of the action potential is largely dependent on Na^+^/Ca^2+^ exchanger activity, the reduced effect of isoproterenol on contraction in myocytes lacking TPC2 proteins was hypothesized to arise from reduced effects on Ca^2+^ transients. This was investigated directly using the Ca^2+^ probe Fluo-5F. Fig. 1, *G* and *H,* shows representative Ca^2+^ transients in myocytes stimulated at 1 Hz. In line with the contraction data, the effects of isoproterenol (3 nm) on Ca^2+^ transients in myocytes from *Tpcn2*^−/−^ mice were significantly reduced compared with WT (maximum increase from control amplitude of 123 ± 24% in WT and 57 ± 17% in *Tpcn2*^−/−^ myocytes, *n* = 15 for both groups, *p* < 0.05, see Fig. 1*I*).

Previous work has demonstrated that NAADP has no effect on Ca^2+^ influx through L-type channels (31, 33). We therefore compared the change in L-type current recorded from *Tpcn2*^−/−^ and WT cells during β-adrenergic stimulation. The effect of 3 nm isoproterenol to increase the amplitude of L-type Ca^2+^ currents remained unchanged in myocytes from *Tpcn2*^−/−^ mice (Fig. 1, *K–M*, 78 ± 5.8% increase in WT and 81 ± 15% increase in *Tpcn2*^−/−^, *p* = 0.88, *n* = 7 for both groups) showing that the β-adrenoreceptor signaling pathway *per se* remained intact, allowing PKA to phosphorylate L-type Ca^2+^ channels in the usual way.

The observations in myocytes from *Tpcn2*^−/−^ mice were complemented by observations in whole hearts perfused by the Langendorff technique. In spontaneously beating hearts, isoproterenol (5 nm) increased the force of ventricular contraction by 92 ± 4% (*n* = 5) in age-matched WT controls compared with 63 ± 9% (*n* = 7) in hearts from *Tpcn2*^−/−^ animals, a significant reduction in animals lacking TPC2 (*p* < 0.05, Fig. 1*J*).

**FIGURE 2.**
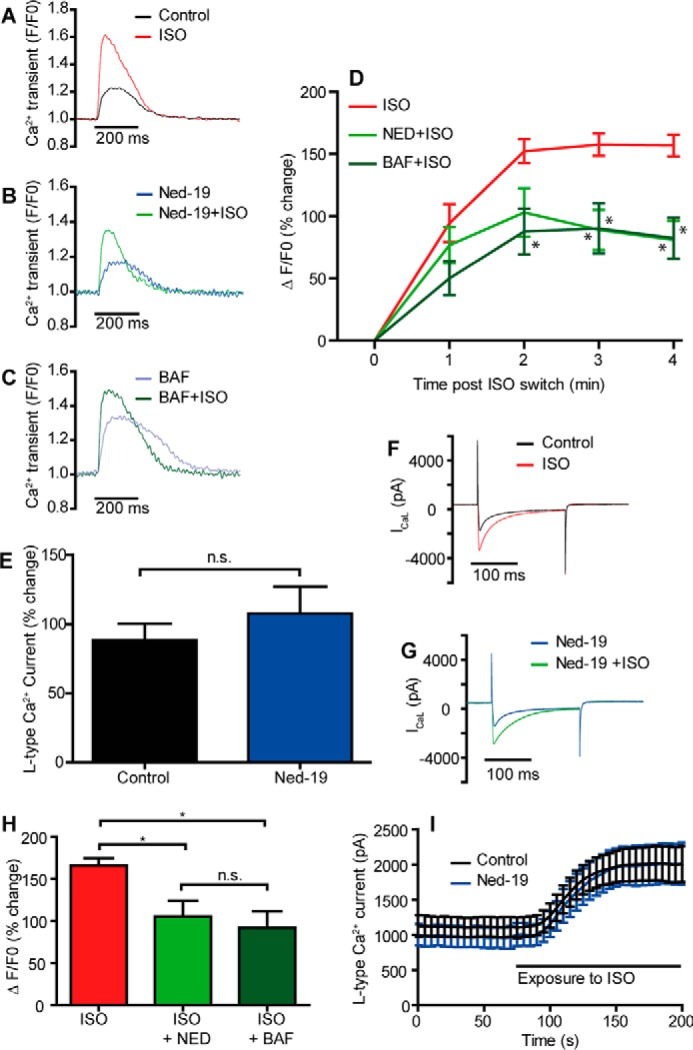
**Effects of β-adrenoreceptor stimulation on Ca^2+^ transients are reduced by suppressing lysosomal function or inhibiting NAADP actions in guinea pig myocytes.** *A–C* show superimposed Ca^2+^ transients (Fluo-5F) in ventricular myocytes (1 Hz stimulation) before and after 2 nm ISO (*A,* control; *B* following 1 μm Ned-19 applied in the patch solution; or *C,* 1 μm bafilomycin (*Baf*)). *D* shows mean data (* indicates significant difference from ISO control condition *p* < 0.05). *E* shows mean data indicating that ISO remained effective in increasing L-type Ca^2+^ currents (with example of superimposed currents before and after ISO in *F* and *G*). *n.s.*, not significant. *H* shows summary graph of maximum change in whole-cell Ca^2+^ transient in guinea pig ventricular myocytes exposed to ISO (2 nm, *n* = 9) or ISO in the presence of either bafilomycin (1 μm, *n* = 5) or Ned-19 (1 μm, *n* = 6). *I* shows a time course of L-type Ca^2+^ current response to ISO (2 nm) under control conditions and in the presence of Ned-19 (both groups *n* = 6). * indicates significant difference between indicated data groups (*p* < 0.05).

The reduced response to isoproterenol in *Tpcn2*^−/−^ myocytes shows a striking parallel to additional observations made during pharmacological suppression of the NAADP pathway in guinea pig ventricular myocytes; exposure to bafilomycin A (1 μm, which abolishes acidic store Ca^2+^ loading (31, 33)) or Ned-19 (1 μm, an antagonist of NAADP (43)) both reduced the Ca^2+^ transient response to isoproterenol (166 ± 8% increase in control cells, *n* = 9, 92 ± 20% increase in the presence of bafilomycin, *n* = 5, and 105 ± 19% increase in the presence of Ned-19, *n* = 6, Fig. 2, *A–D* and *H*). Although suppression of the NAADP pathway reduced the ability of isoproterenol to increase Ca^2+^ transients, the effect of isoproterenol on L-type Ca^2+^ currents was unaffected by Ned-19 showing that other stages of the β-adrenoreceptor pathway remained operational (Fig. 2, *E–G* and *I*).

Taken together, the observations reported above provide a compelling case for the contention that NAADP, most likely acting via the TPC2 protein, plays a major role in the acute effects of isoproterenol on the magnitude of stimulated Ca^2+^ transients and contractions in ventricular myocytes from mouse and guinea pig.

**FIGURE 3.**
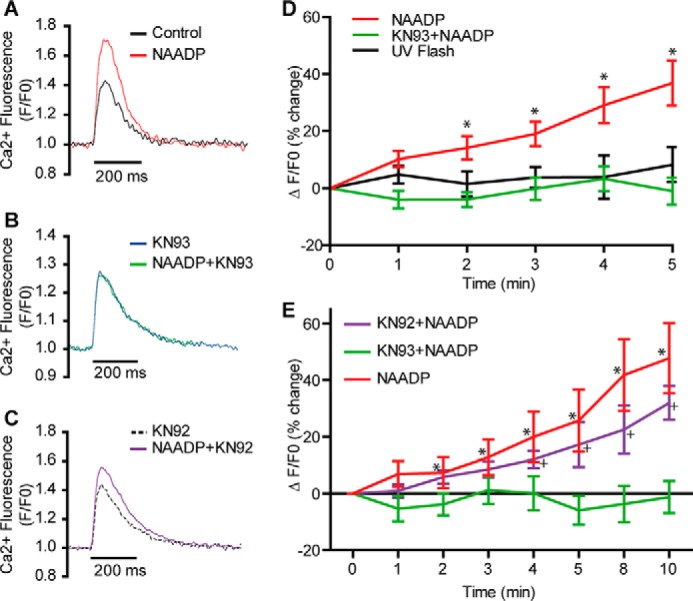
**CaMKII is required for atrial cardiomyocyte responses to NAADP from guinea pig.** *A* and *B* show superimposed representative Ca^2+^ transients (Fluo-5F, 1 Hz) before and after photorelease of NAADP from a caged NPE derivative in the absence (*A*) or presence (*B*) of 1 μm KN-93 or (*C*) 1 μm KN-92 (a structural analogue of KN-93 lacking effects on CaMKII) in guinea pig atrial myocytes; *D* shows mean data (*n* = 10 control, *n* = 6 KN-93). Note suppression by KN-93 of effect of photoreleased NAADP on Ca^2+^ transients. *E* shows mean data comparing KN-92 and KN-93 over 10 min (*n* = 4 for each). Note lack of effect of KN-92 in comparison to KN-93. *, *p* < 0.05.

Previous work has shown that the effects of NAADP to increase the amplitude of Ca^2+^ transients triggered by action potentials are associated with an increased Ca^2+^ load of the SR (31, 33). This increased Ca^2+^ load provides a major mechanism for the increased amplitude of the Ca^2+^ transient accompanying Ca^2+^-induced Ca^2+^ release, although additional effects of NAADP to influence the “gain” of Ca^2+^-induced Ca^2+^ release cannot be excluded. Does the reported increase in SR Ca^2+^ load in response to NAADP result solely from the small amounts of Ca^2+^ released from lysosomes or might there be an amplification mechanism? One possibility is that Ca^2+^ released from the lysosome could activate CaMKII causing phosphorylation of phospholamban and disinhibition of SERCA activity resulting in additional SR Ca^2+^ load (44). This was tested in guinea pig myocytes by employing two CaMKII inhibitors (KN-93, 1 μm, in atrial myocytes and AIP peptide, 1 μm, in ventricular myocytes). Following photorelease of NAADP from a caged NPE derivative (40), there were increases in the amplitude of Ca^2+^ transients in guinea pig atria (37 ± 8% increase after 5 min, *n* = 10) similar to those described previously (Fig. 3, *A, D,* and *E*) (31, 33). These effects of photoreleased NAADP on Ca^2+^ transients were abolished by KN-93 (Fig. 3, *B, D,* and *E*). KN-92 is a structurally related analogue of KN-93 without effects on CaMKII, and this substance failed to suppress the effects of photoreleased NAADP in atrial myocytes (Fig. 3, *C* and *E*).

**FIGURE 4.**
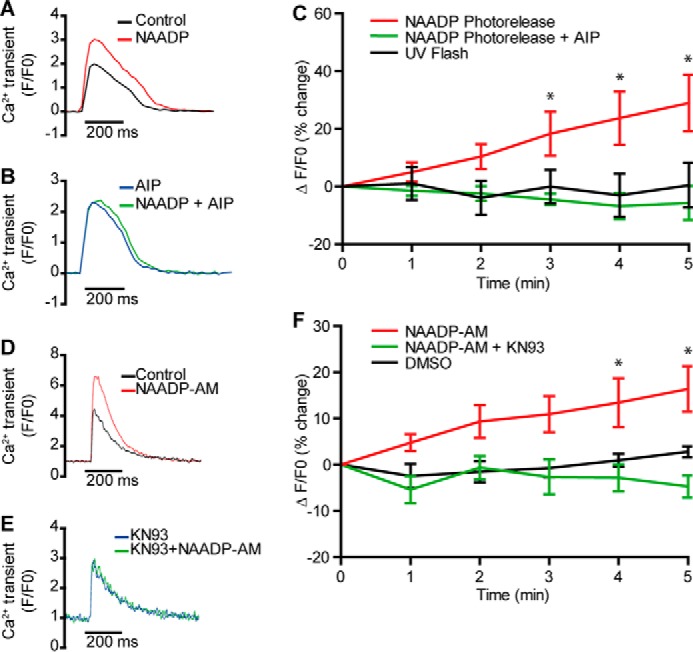
**CaMKII is required for ventricular cardiomyocyte responses to NAADP from guinea pig or WT mice.** *A* and *B* show traces from similar experiments in guinea pig ventricular myocytes in the absence (*A*) and presence (*B*) of inhibitor peptide (AIP, 1 μm in the patch solution, Fluo5F, 1 Hz stimulation); *C* shows mean data (*n* = 8 control, *n* = 7 AIP). *D* and *E* show Ca^2+^ transients (Fluo-5F, 1 Hz) in mouse ventricular myocytes before after application of NAADP-AM in the absence (*D*) and presence (*E*) of 1 μm KN93. *F* shows mean data (*n* = 20 for NAADP-AM, *n* = 10 for KN93, and *n* = 5 for DMSO controls). * indicates significant difference from 0-min value *p* < 0.05.

Effects of the active KN-93 were also tested in ventricular myocytes from WT mice exposed to NAADP-AM. Again, Ca^2+^ transients were increased following exposure to NAADP-AM in the absence (*n* = 20) but not the presence of KN-93 (*n* = 10, Fig. 4, *D–F*). Similar observations were made in guinea pig ventricular myocytes (Fig. 4, *A–C*, in which the highly selective CaMKII inhibitor, AIP, suppressed the effects of photoreleased NAADP (38 ± 9% increase in the amplitude at 5 min after photorelease of NAADP under control conditions, *n* = 8, but a 6 ± 5% decrease in amplitude 5 min after photorelease of NAADP in the presence of AIP, *n* = 7)). These observations are therefore consistent with a role for CaMKII in the pathway-mediating effects of NAADP in guinea pig and mouse myocytes, in part through phospholamban/SERCA, although additional effects, for example on ryanodine receptors, cannot be excluded.

**FIGURE 5.**
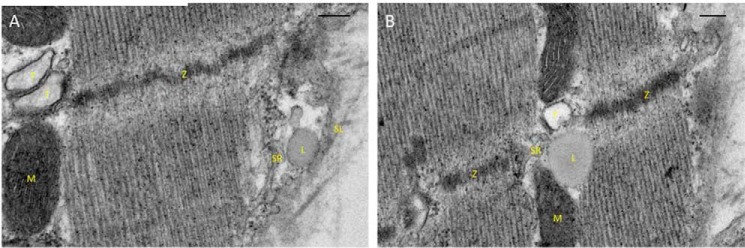
**Electron micrographs showing the subcellular relationships of cardiac lysosomes in rabbit.** Lysosomes were identified according to their well established appearance and features (*i.e.* a granular and approximately uniform matrix that has a higher electron density than the surrounding cytoplasm, bounded by a single lipid bilayer membrane). *A* shows a lysosome closely apposed to SR but some distance from the Z-line and t-tubules. In contrast, *B* shows a lysosome (*L*) near a Z-line (*Z*) and in close apposition with the sarcoplasmic reticulum (*SR*) and a t-tubule (*T*). Mitochondria (*M*) can also be seen in both panels, and the sarcolemma (*SL*) can be seen in *A*. These images suggest the existence of lysosomes that are adjacent to structures associated with Ca^2+^ reuptake and release, respectively. *Scale bars,* 200 nm.

For an amplification mechanism to work in the way we have hypothesized above, a close structural relationship would be required to exist between lysosomes and the SR. This was investigated using transmission electron microscopy of rabbit ventricular tissue, and sample images are shown in Fig. 5. It can be seen that lysosomes were observed close to the SR, both in regions that might be associated with Ca^2+^ uptake (Fig. 5*A*) and in locations thought to mediate Ca^2+^ release via RyRs (Fig. 5*B*). The mean distance of lysosomes from the SR was 25.4 ± 1.8 nm (*n* = 30). The significance of these observations is considered in more detail under the “Discussion.”

**FIGURE 6.**
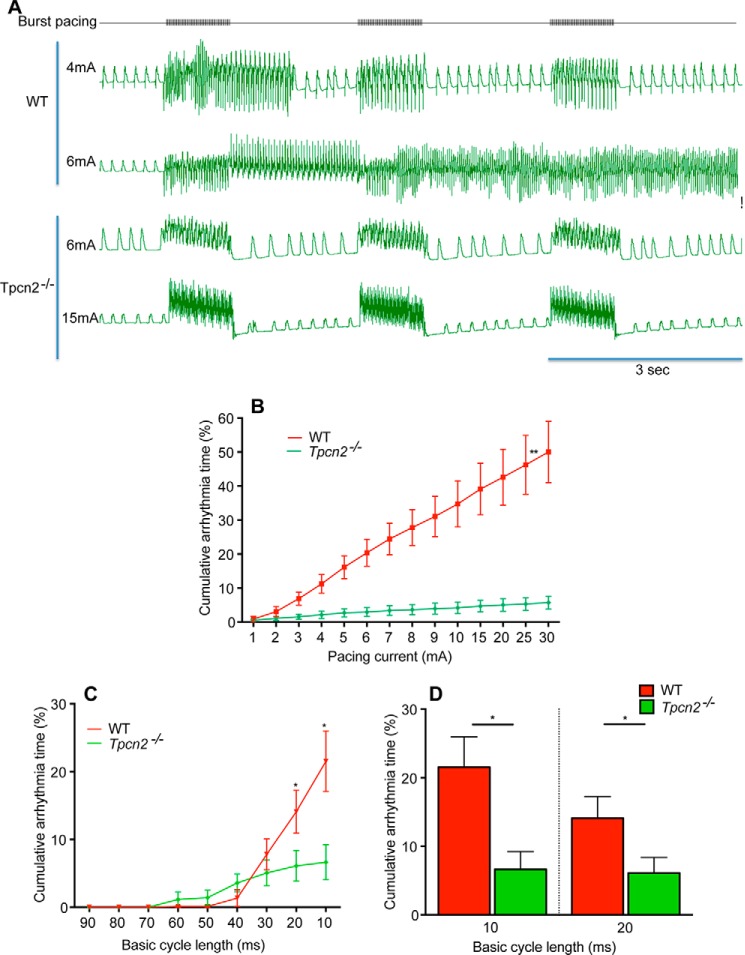
**Isolated whole hearts from *Tpcn2*^−/−^ mice are less prone to arrhythmias induced by acute and high dose isoproterenol exposure (50 nm).** The susceptibility to arrhythmias was investigated using a ventricular burst pacing protocol in which three periods of stimulation (1-s train) were applied with 2-s intervals, whereas monophasic action potentials were recorded. Representative recordings are shown in *A*. Cumulative arrhythmia time represents the percentage of time showing arrhythmias (ventricular tachycardia or ventricular fibrillation) after burst pacing (measured in the 2-s intervals following stimulation). The effect of increasing stimulation amplitude is shown in *B* and that of reducing cycle length in *C* and *D*. **, *p* < 0.001; *, *p* < 0.05, *n* = 9 for WT group, *n* = 10 for *Tpcn2*^−/−^ group.

From these observations showing that NAADP and TPC2 appear to participate in the effects of the β-adrenoreceptor agonist isoproterenol to increase the amplitude of Ca^2+^ transients in electrically stimulated myocytes, and taking into account the finding that arrhythmias caused by isoproterenol could be suppressed by the NAADP antagonist BZ194 (34), we hypothesized that isoproterenol-mediated increases in NAADP acting via TPC2 proteins might also be involved in the susceptibility to cardiac arrhythmias in the intact heart. We first investigated acute exposure to the β-adrenoreceptor agonist. In the presence of isoproterenol, hearts from *Tpcn2*^−/−^ mice were significantly (*p* < 0.001) less prone to arrhythmias when subjected to a ventricular burst pacing protocol compared with WT controls. The induction threshold for ventricular tachycardia or fibrillation using a burst pacing protocol in hearts exposed to isoproterenol was significantly higher in hearts from *Tpcn2*^−/−^ mice compared with hearts from WT mice (Fig. 6).

**FIGURE 7.**
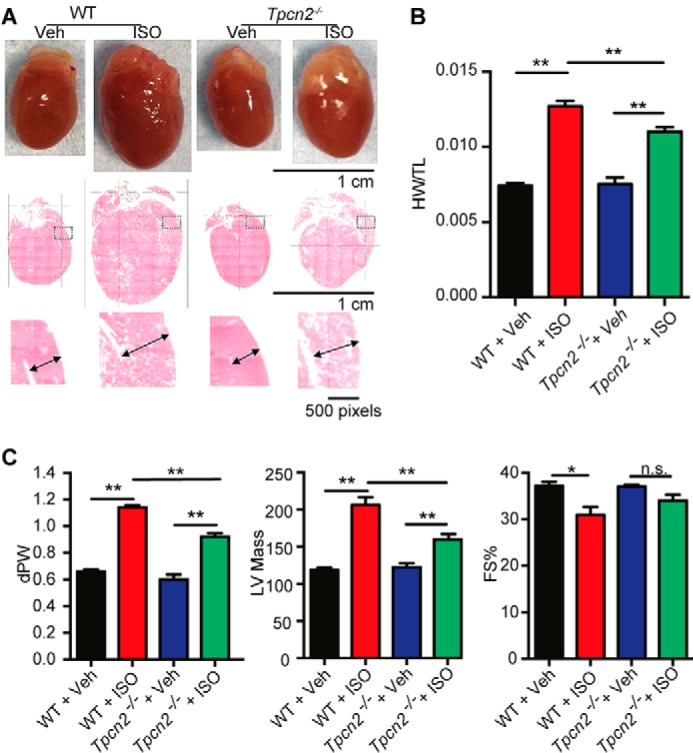
***Tpcn2*^−/−^ mice exhibit less hypertrophy and better cardiac function than wild-type mice after chronic β-adrenergic stimulation for 14 days *in vivo* by osmotic mini-pump.** The reduced hypertrophy after chronic ISO in *Tpcn2*^−/−^ mice is illustrated in *A*, showing whole hearts and representative examples of morphometry (example of cardiac slices and magnified regions in which *double-headed arrows* indicate ventricular wall thickness). *Veh*, vehicle. *B* shows hypertrophy indicated by a comparison of mean heart weight (*HW*)/tibia length (*TL*) ratios. *C* shows echocardiography data (*dPW*, end diastolic left ventricular posterior wall thickness; *LV mass*, left ventricular mass; and *FS%,* reduction of fractional shortening), again illustrating the reduced hypertrophy in hearts from *Tpcn2*^−/−^ mice compared with WT. *n.s.*, not significant; *, *p* < 0.05; **, *p* < 0.01.

**FIGURE 8.**
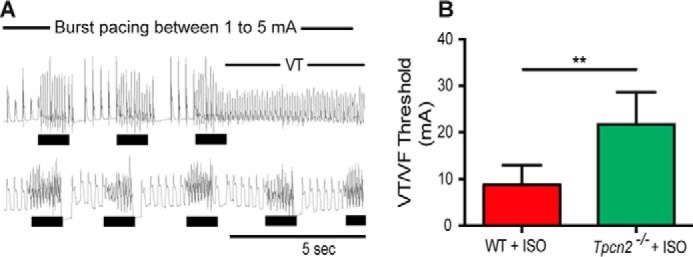
**Hearts from *Tpcn2*^−/−^ mice exposed to chronic β-adrenergic stimulation for 14 days *in vivo* by osmotic mini-pump show lower susceptibility to ventricular arrhythmias than hearts from wild-type mice.** *A* shows representative examples of monophasic action potential recordings in Langendorff-perfused hearts after exposure to chronic ISO stress (ventricular burst pacing with a train of 50 S1 at a cycle length of 20 ms at a progressively increasing amplitude of pacing current), whereas *B* illustrates the increased threshold for induction of ventricular tachycardia/fibrillation by burst pacing in *Tpcn2*^−/−^ mice. Data are presented as means ± S.E. **, *p* < 0.01. *n* = 6 for each group.

Building upon the above observations showing a role for NAADP and TPC2 proteins in the acute effects of β-adrenoreceptor stimulation and acute arrhythmogenesis, we investigated whether NAADP-mediated Ca^2+^ signaling may also play a role in the development of hypertrophy that is caused by chronic stimulation of β-adrenoceptors. To test this hypothesis, both *Tpcn2*^−/−^ and WT mice were stressed by chronic administration of isoproterenol at 10 mg/kg/day for 14 days via osmotic mini-pumps. The control groups were given isotonic saline following the same protocol. Notably, after 14 days of treatment with isoproterenol, *Tpcn2*^−/−^ mice displayed less hypertrophy and showed better preserved cardiac function than WT mice as demonstrated by echocardiography, morphometry, and histology (Fig. 7, *A–C*). In particular, the increase in heart weight after chronic exposure to isoproterenol (expressed either as left ventricular mass or the ratio of heart weight to tibia length) was significantly less in *Tpcn2*^−/−^ mice than in WT (heart weight/tibia length after 14 days of isoproterenol treatment 0.0127 ± 0.0004 g mm^−1^ in wild type against 0.0110 ± 0.0003 g mm^−1^ in *Tpcn2*^−/−^, *p* < 0.05). Additional *ex vivo* electrophysiological studies indicated that hearts from isoproterenol-treated *Tpcn2*^−/−^ mice showed less susceptibility to arrhythmia when subjected to a ventricular burst pacing protocol in the absence of further acute isoproterenol exposure (Fig. 8, *A* and *B*, with threshold currents required to elicit ventricular fibrillation increasing from 8.8 ± 4.2 to 21.7 ± 6.9 mA, *p* < 0.05).

## Discussion

The observations described in this study demonstrate the requirement for TPC2 proteins in order for NAADP actions in mammalian cardiac tissue, and they highlight the contribution of these proteins during acute and chronic actions of the β-adrenergic signaling pathway. We have shown by using both genetic and pharmacological techniques that the NAADP pathway is physiologically important during acute β-adrenergic stimulation. Furthermore, pharmacological observations show that the actions of NAADP on cellular Ca^2+^ signaling require the presence of functional CaMKII. TPC2 proteins also appear to contribute to arrhythmogenic effects during excessive acute β-adrenergic stimulation. In addition, our work highlights the contribution of this pathway during chronic β-adrenergic stimulation leading to pathological hypertrophy and suggests that its inhibition is partially protective against the negative consequences of chronic activation of β-adrenergic signaling pathways.

The role of two-pore channels in the generation of Ca^2+^ signals is an emerging field. Ca^2+^ release from lysosomes dependent on TPC2 has been demonstrated to regulate a disparate number of cellular processes, and it is the principal way in which NAADP evokes intracellular Ca^2+^ signals. Roles for TPCs in pathophysiological processes to date include triggering of the acrosome reaction in mammalian fertilization (20), angiogenesis (23), cell differentiation (45–47), muscarinic receptor-mediated smooth muscle contraction (27), endocytic trafficking of cholesterol in the liver (25), insulin secretion from mouse pancreatic β-cells (21), T cell activation (48), exocrine pancreatic Ca^2+^ signaling (24), melanosome concentration in *Xenopus* oocytes (26), defective vesicular trafficking in mouse models of Parkinson disease (49), and Ebola infectivity (19). The overriding principle of NAADP-evoked Ca^2+^ signaling is that localized Ca^2+^ signals are evoked by the release of lysosomal Ca^2+^. This has three major roles. Localized Ca^2+^ release in the lysosomal system promotes vesicular trafficking and also exocytosis, whereas localized sub-plasma membrane release modulates membrane excitability (18). However, a major role for NAADP is to enhance the excitability of the ER by triggering Ca^2+^-induced Ca^2+^ release leading to global Ca^2+^ signals at lysosomal-ER nanojunctions (18).

The slow time course of the increase in Ca^2+^ signals following photorelease of NAADP led us to hypothesize that there may be more to the observed effects than the simple two-pool model (3) in which Ca^2+^ rapidly released from lysosomes is taken up into the ER/SR and leads to increased ER/SR release during Ca^2+^-induced Ca^2+^ release. Whether such communication involves an increase in cytosolic peri-SR Ca^2+^ to activate inositol 1,4,5-trisphosphate receptors or RyRs or whether a local increase in luminal SR Ca^2+^ is required is not clear.

In this study we have shown that CaMKII is necessary to mediate the effects of NAADP. We suggest that CaMKII may amplify the effect of Ca^2+^ released from acidic lysosomes, leading to phosphorylation of proteins on the SR membrane. Our previous observations showing that NAADP actions in the heart are associated with an increased amount of Ca^2+^ loaded into the SR led us to suggest that the major actions of CaMKII-mediated phosphorylation are likely to occur from relief of phospholamban-mediated inhibition of SERCA (31, 33), but additional actions on, for example, the ryanodine receptor (that might also be prevented by CaMKII inhibition) cannot be excluded.

**FIGURE 9.**
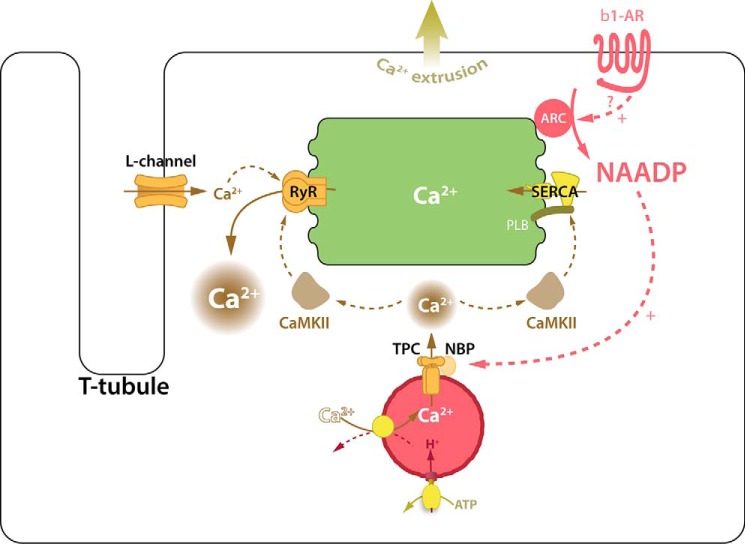
**Proposed scheme for actions of NAADP in cardiac myocytes.** Scheme for action of NAADP in cardiac myocytes consistent with published observations with the addition of our present results. Activation of β-adrenoceptors causes elevation of cellular NAADP levels (probably by increasing the activity of an ADP-ribosyl-cyclase). NAADP acts to release Ca^2+^ from lysosomes through the TPC2 channel. The Ca^2+^ released from the acidic store via TPC2 leads to enhancement of the Ca^2+^ transient triggered by Ca^2+^ entry through L-type Ca^2+^ channels during cardiac action potentials. This enhancement of the Ca^2+^ transient may result from effects of Ca^2+^ released from the acidic store on CaMKII (increasing the amount of Ca^2+^ loaded into the SR or perhaps increasing the Ca^2+^ sensitivity of RyRs). The abbreviations used are as follows: β*-AR,* β-adrenoreceptor; *L-channel*, L-type voltage-gated Ca^2+^ channel; *PLB*, phospholamban.

A scheme summarizing our proposals is shown in Fig. 9. Future experiments will be needed to further test this hypothesis. In particular, it will be important to investigate whether there is a preferential location of lysosomes/late endosomes bearing TPC2 proteins. It has been acknowledged for many years that close associations between subcellular structures known as nanojunctions or microdomains play an important role in localized Ca^2+^ signaling. Membrane contact sites are vital to the formation of functional signaling microdomains and are regions where membranes typically come within 30 nm of each other. This small space allows the release of a small amount of Ca^2+^ to result in a high concentration and therefore more efficient and rapid signaling. Examples include the t-tubule membrane and the SR (allowing close proximity between L-type Ca^2+^ channels and ryanodine receptors) and also the mitochondrial membrane and the SR (allowing a similar relationship between inositol 1,4,5-trisphosphate receptors and mitochondrial Ca^2+^ transporters). These relationships are not simply a structural coincidence; they not only facilitate major physiological processes, such as excitation-contraction coupling and regulation of oxidative phosphorylation respectively, but recent work has suggested molecular tethering between specific proteins on these structures to maintain their close apposition (50, 51).

Such microdomains have been observed in extracardiac tissues between lysosomes and the SR/ER (52). In pulmonary artery smooth muscle cells NAADP-dependent Ca^2+^ release was observed principally in the perinuclear region in association with RyR3, which colocalized with lysosomal/late endosomal markers such as LAMP-1 (1).

Our results suggest that these lysosome-SR microdomains also exist in cardiac tissue. In the future, it will be important to investigate whether there is a preferential location of lysosomes that bear TPC2 proteins in the heart, as this would indicate where microdomains involved in NAADP-related Ca^2+^ signaling are likely to be located. Although our observations of lysosomes using transmission electron microscopy were not sufficient to provide information regarding the exact distribution of TPC2 proteins, they do provide good evidence that lysosomes are located in positions that are consistent with our hypothesis. Those lysosomes that are closely associated with t-tubules might be expected to facilitate effects of NAADP on Ca^2+^ release from the SR via RyR2. Similarly, those lysosomes observed to be more distant from the t-tubules could be associated with SERCA and phospholamban or with CaMKII and be involved with the facilitation of Ca^2+^ reuptake and increased SR Ca^2+^ load (although SERCA may be more widely distributed in SR membranes).

The proposed novel effects of CaMKII as part of the cardiac NAADP signaling pathway provide an additional mechanism for the extensive contributions of CaMKII to β-adrenoreceptor signaling in the heart (53). A role for CaMKII has frequently been associated with cardiac hypertrophy (54–57) and arrhythmogenesis (58), and the pathway involving NAADP and TPC2 proposed here might provide insight into a novel additional mechanism by which such effects could arise.

Our observations can be readily incorporated in the accepted scheme that β-adrenoreceptor agonists lead to stimulation of adenylyl cyclase, formation of cAMP, and activation of PKA, followed by phosphorylation of proteins, including L-type Ca^2+^ channels (to promote Ca^2+^ entry) and phospholamban (speeding relaxation and increasing the amount of Ca^2+^ stored in the SR). To this scheme, we would add that NAADP synthesis is also increased during β-adrenergic stimulation (31, 32). A family of multifunctional enzymes termed ADP-ribosyl cyclases has been suggested to synthesize both cADP-ribose (when the substrate is NAD) and NAADP (when the substrate is NADP with nicotinic acid). These enzymes may be located in the sarcolemma but have also been suggested to be associated with the SR (59, 60). Given that NAADP synthesis has been shown to be stimulated by activation of β-adrenergic receptors, it seems plausible that these enzymes might be an additional target of phosphorylation by PKA (59, 61, 62) or as a consequence of other coupling mechanisms that have yet to be elucidated. The resultant NAADP could act on TPC2 proteins in acidic lysosomes to bring about effects that supplement other well established effects of β-adrenoreceptor stimulation on L-type Ca^2+^ currents, RyR, and phospholamban. PKA-mediated phosphorylation of phospholamban is a major mechanism underlying the increased Ca^2+^ load of the SR following β-adrenoreceptor stimulation (58, 63), although there may be an additional contribution arising from phosphorylation by CaMKII (44, 53, 54). The reported actions of NAADP to increase cardiac muscle Ca^2+^ transients and contraction are also associated with an increase in SR Ca^2+^ load in both atrial and ventricular myocytes (31, 33).

The mechanisms underlying cardiac hypertrophy following chronic β-adrenoreceptor stimulation are likely to be complex, but the evidence reported here supports a role for TPC2 proteins.

In summary, our observations provide strong support for a role for NAADP and TPC2 proteins as major contributors in the actions of β-adrenoreceptor agonists to increase contraction amplitude in cardiac ventricular myocytes and suggest that these effects require the presence of functional CaMKII. TPC2 proteins also appear to play a major role in the pathway linking the stress of chronic stimulation of β-adrenoceptors to hypertrophy and associated tendency to arrhythmias. These proteins are likely to provide a promising new target for future therapy, because inhibiting the NAADP/TPC2 pathway has minimal effect on normal excitation-contraction coupling but suppresses adrenergic signaling in the heart.

## Author Contributions

R. A. C. carried out all guinea pig work; E. L. B. and W. K. L. carried out all *Tpcn2*^−/−^ physiology; cell isolations were carried out by E. L. B., R. A. C., and W. K. L. W. K. L. synthesized NAADP-AM; D. B-Y. and G. C. C. synthesized caged NAADP. M. R., J. P., and A. G. were responsible for the development of the *Tpcn2*^−/−^ mouse line and subsequent breeding. K. T. S. carried out confirmation of *Tpcn2*^−/−^ mRNA. R. A. B. B. and D. A. performed electron microscopy studies. A. G., M. R., and D. A. T. were responsible for experimental design, with assistance from R. A. C., E. L. B., W. K. L., and X. W. W. L. carried out echocardiography. D. A. carried out experiments on arrhythmias during acute isoproterenol stimulation. Y. W. carried out osmotic minipump experiments and chronic isoproterenol stimulation work. All experiments were carried out in the laboratories of D. A. T. and M. R. All authors contributed to the writing and editing of the manuscript.

## Footnotes

11 The abbreviations used are:

NAADP: nicotinic acid adenine dinucleotide phosphate
SR: sarcoplasmic reticulum
TPC: two pore channel
CaMKII: Ca^2+^/calmodulin-dependent protein kinase II
ER: endoplasmic reticulum
SERCA: sarco/endoplasmic reticulum Ca^2+^-ATPase
RyR: ryanodine receptor
PSS: physiological salt solution
ISO: isoproterenol
PES: programmed electrical stimulation
NPE: 1(2-nitrophenyl)diazoethane
AIP: autocamtide-2-related inhibitory peptide.

## References

[1] Kinnear N. P., Boittin F. X., Thomas J. M., Galione A., and Evans A. M. (2004) Lysosome-sarcoplasmic reticulum junctions: a trigger zone for calcium signaling by nicotinic acid adenine dinucleotide phosphate and endothelin-1. J. Biol. Chem. 279, 54319–54326 15331591 10.1074/jbc.M406132200

[2] Lee H. C. (2001) Physiological functions of cyclic ADP-ribose and NAADP as calcium messengers. Annu. Rev. Pharmacol. Toxicol. 41, 317–345 11264460 10.1146/annurev.pharmtox.41.1.317

[3] Churchill G. C., and Galione A. (2001) NAADP induces Ca^2+^ oscillations via a two-pool mechanism by priming IP3- and cADPR-sensitive Ca^2+^ stores. EMBO J. 20, 2666–2671 11387201 10.1093/emboj/20.11.2666 PMC125473

[4] Churchill G. C., Okada Y., Thomas J. M., Genazzani A. A., Patel S., and Galione A. (2002) NAADP mobilizes Ca^2+^ from reserve granules, lysosome-related organelles, in sea urchin eggs. Cell 111, 703–708 12464181 10.1016/s0092-8674(02)01082-6

[5] Morgan A. J., and Galione A. (2007) NAADP induces pH changes in the lumen of acidic Ca^2+^ stores. Biochem. J. 402, 301–310 17117921 10.1042/BJ20060759 PMC1798430

[6] Morgan A. J., Platt F. M., Lloyd-Evans E., and Galione A. (2011) Molecular mechanisms of endolysosomal Ca^2+^ signalling in health and disease. Biochem. J. 439, 349–374 21992097 10.1042/BJ20110949

[7] Yamasaki M., Masgrau R., Morgan A. J., Churchill G. C., Patel S., Ashcroft S. J., and Galione A. (2004) Organelle selection determines agonist-specific Ca^2+^ signals in pancreatic acinar and β cells. J. Biol. Chem. 279, 7234–7240 14660554 10.1074/jbc.M311088200

[8] Zhang F., Zhang G., Zhang A. Y., Koeberl M. J., Wallander E., and Li P. L. (2006) Production of NAADP and its role in Ca^2+^ mobilization associated with lysosomes in coronary arterial myocytes. Am. J. Physiol. Heart Circ. Physiol. 291, H274–H282 16473958 10.1152/ajpheart.01064.2005

[9] Calcraft P. J., Ruas M., Pan Z., Cheng X., Arredouani A., Hao X., Tang J., Rietdorf K., Teboul L., Chuang K. T., Lin P., Xiao R., Wang C., Zhu Y., Lin Y., et al (2009) NAADP mobilizes calcium from acidic organelles through two-pore channels. Nature 459, 596–600 19387438 10.1038/nature08030 PMC2761823

[10] Patel S., Marchant J. S., and Brailoiu E. (2010) Two-pore channels: regulation by NAADP and customized roles in triggering calcium signals. Cell Calcium 47, 480–490 20621760 10.1016/j.ceca.2010.05.001 PMC2921607

[11] Ruas M., Davis L. C., Chen C. C., Morgan A. J., Chuang K. T., Walseth T. F., Grimm C., Garnham C., Powell T., Platt N., Platt F. M., Biel M., Wahl-Schott C., Parrington J., and Galione A. (2015) Expression of Ca^2+^-permeable two-pore channels rescues NAADP signalling in TPC-deficient cells. EMBO J. 34, 1743–1758 25872774 10.15252/embj.201490009 PMC4516428

[12] Zong X., Schieder M., Cuny H., Fenske S., Gruner C., Rötzer K., Griesbeck O., Harz H., Biel M., and Wahl-Schott C. (2009) The two-pore channel TPCN2 mediates NAADP-dependent Ca^2+^-release from lysosomal stores. Pflugers Arch. 458, 891–899 19557428 10.1007/s00424-009-0690-y PMC2719734

[13] Patel S. (2015) Function and dysfunction of two-pore channels. Sci. Signal. 8, re7 26152696 10.1126/scisignal.aab3314

[14] Churamani D., Hooper R., Brailoiu E., and Patel S. (2012) Domain assembly of NAADP-gated two-pore channels. Biochem. J. 441, 317–323 21992073 10.1042/BJ20111617 PMC3242506

[15] Rietdorf K., Funnell T. M., Ruas M., Heinemann J., Parrington J., and Galione A. (2011) Two-pore channels form homo- and heterodimers. J. Biol. Chem. 286, 37058–37062 21903581 10.1074/jbc.C111.289835 PMC3199452

[16] Lin-Moshier Y., Walseth T. F., Churamani D., Davidson S. M., Slama J. T., Hooper R., Brailoiu E., Patel S., and Marchant J. S. (2012) Photoaffinity labeling of nicotinic acid adenine dinucleotide phosphate (NAADP) targets in mammalian cells. J. Biol. Chem. 287, 2296–2307 22117075 10.1074/jbc.M111.305813 PMC3268391

[17] Walseth T. F., Lin-Moshier Y., Jain P., Ruas M., Parrington J., Galione A., Marchant J. S., and Slama J. T. (2012) Photoaffinity labeling of high affinity nicotinic acid adenine dinucleotide phosphate (NAADP)-binding proteins in sea urchin egg. J. Biol. Chem. 287, 2308–2315 22117077 10.1074/jbc.M111.306563 PMC3268392

[18] Galione A. (2015) A primer of NAADP-mediated Ca signalling: From sea urchin eggs to mammalian cells. Cell Calcium 58, 27–47 25449298 10.1016/j.ceca.2014.09.010

[19] Sakurai Y., Kolokoltsov A. A., Chen C. C., Tidwell M. W., Bauta W. E., Klugbauer N., Grimm C., Wahl-Schott C., Biel M., and Davey R. A. (2015) Ebola virus. Two-pore channels control Ebola virus host cell entry and are drug targets for disease treatment. Science 347, 995–998 25722412 10.1126/science.1258758 PMC4550587

[20] Arndt L., Castonguay J., Arlt E., Meyer D., Hassan S., Borth H., Zierler S., Wennemuth G., Breit A., Biel M., Wahl-Schott C., Gudermann T., Klugbauer N., and Boekhoff I. (2014) NAADP and the two-pore channel protein 1 participate in the acrosome reaction in mammalian spermatozoa. Mol. Biol. Cell 25, 948–964 24451262 10.1091/mbc.E13-09-0523 PMC3952862

[21] Arredouani A., Ruas M., Collins S. C., Parkesh R., Clough F., Pillinger T., Coltart G., Rietdorf K., Royle A., Johnson P., Braun M., Zhang Q., Sones W., Shimomura K., Morgan A. J., et al (2015) NAADP and endolysosomal two-pore channels modulate membrane excitability and stimulus-secretion coupling in mouse pancreatic beta cells. J. Biol. Chem. 290, 21376–21392 26152717 10.1074/jbc.M115.671248 PMC4571866

[22] Cang C., Zhou Y., Navarro B., Seo Y. J., Aranda K., Shi L., Battaglia-Hsu S., Nissim I., Clapham D. E., and Ren D. (2013) mTOR regulates lysosomal ATP-sensitive two-pore Na^+^ channels to adapt to metabolic state. Cell 152, 778–790 23394946 10.1016/j.cell.2013.01.023 PMC3908667

[23] Favia A., Desideri M., Gambara G., D'Alessio A., Ruas M., Esposito B., Del Bufalo D., Parrington J., Ziparo E., Palombi F., Galione A., and Filippini A. (2014) VEGF-induced neoangiogenesis is mediated by NAADP and two-pore channel-2-dependent Ca^2+^ signaling. Proc. Natl. Acad. Sci. U.S.A. 111, E4706–4715 25331892 10.1073/pnas.1406029111 PMC4226099

[24] Gerasimenko J. V., Charlesworth R. M., Sherwood M. W., Ferdek P. E., Mikoshiba K., Parrington J., Petersen O. H., and Gerasimenko O. V. (2015) Both RyRs and TPCs are required for NAADP-induced intracellular Ca release. Cell Calcium 58, 237–245 26100948 10.1016/j.ceca.2015.05.005 PMC4539342

[25] Grimm C., Holdt L. M., Chen C. C., Hassan S., Müller C., Jörs S., Cuny H., Kissing S., Schröder B., Butz E., Northoff B., Castonguay J., Luber C. A., Moser M., Spahn S., et al (2014) High susceptibility to fatty liver disease in two-pore channel 2-deficient mice. Nat. Commun. 5, 4699 25144390 10.1038/ncomms5699

[26] Lin-Moshier Y., Keebler M. V., Hooper R., Boulware M. J., Liu X., Churamani D., Abood M. E., Walseth T. F., Brailoiu E., Patel S., and Marchant J. S. (2014) The two-pore channel (TPC) interactome unmasks isoform-specific roles for TPCs in endolysosomal morphology and cell pigmentation. Proc. Natl. Acad. Sci. U.S.A. 111, 13087–13092 25157141 10.1073/pnas.1407004111 PMC4246952

[27] Tugba Durlu-Kandilci N., Ruas M., Chuang K. T., Brading A., Parrington J., and Galione A. (2010) TPC2 proteins mediate nicotinic acid adenine dinucleotide phosphate (NAADP)- and agonist-evoked contractions of smooth muscle. J. Biol. Chem. 285, 24925–24932 20547763 10.1074/jbc.M110.129833 PMC2915728

[28] Wang X., Zhang X., Dong X. P., Samie M., Li X., Cheng X., Goschka A., Shen D., Zhou Y., Harlow J., Zhu M. X., Clapham D. E., Ren D., and Xu H. (2012) TPC proteins are phosphoinositide-activated sodium-selective ion channels in endosomes and lysosomes. Cell 151, 372–383 23063126 10.1016/j.cell.2012.08.036 PMC3475186

[29] Tsaih S. W., Holl K., Jia S., Kaldunski M., Tschannen M., He H., Andrae J. W., Li S. H., Stoddard A., Wiederhold A., Parrington J., Ruas da Silva M., Galione A., Meigs J., Meta-Analyses of Glucose and Insulin-Related Traits Consortium (MAGIC) Investigators, et al (2014) Identification of a novel gene for diabetic traits in rats, mice, and humans. Genetics 198, 17–29 25236446 10.1534/genetics.114.162982 PMC4174929

[30] Lin P. H., Duann P., Komazaki S., Park K. H., Li H., Sun M., Sermersheim M., Gumpper K., Parrington J., Galione A., Evans A. M., Zhu M. X., and Ma J. (2015) Lysosomal two-pore channel subtype 2 (TPC2) regulates skeletal muscle autophagic signaling. J. Biol. Chem. 290, 3377–3389 25480788 10.1074/jbc.M114.608471 PMC4319008

[31] Macgregor A., Yamasaki M., Rakovic S., Sanders L., Parkesh R., Churchill G. C., Galione A., and Terrar D. A. (2007) NAADP controls cross-talk between distinct Ca^2+^ stores in the heart. J. Biol. Chem. 282, 15302–15311 17387177 10.1074/jbc.M611167200

[32] Lewis A. M., Aley P. K., Roomi A., Thomas J. M., Masgrau R., Garnham C., Shipman K., Paramore C., Bloor-Young D., Sanders L. E., Terrar D. A., Galione A., and Churchill G. C. (2012) β-Adrenergic receptor signaling increases NAADP and cADPR levels in the heart. Biochem. Biophys. Res. Commun. 427, 326–329 22995315 10.1016/j.bbrc.2012.09.054

[33] Collins T. P., Bayliss R., Churchill G. C., Galione A., and Terrar D. A. (2011) NAADP influences excitation-contraction coupling by releasing calcium from lysosomes in atrial myocytes. Cell Calcium 50, 449–458 21906808 10.1016/j.ceca.2011.07.007

[34] Nebel M., Schwoerer A. P., Warszta D., Siebrands C. C., Limbrock A. C., Swarbrick J. M., Fliegert R., Weber K., Bruhn S., Hohenegger M., Geisler A., Herich L., Schlegel S., Carrier L., Eschenhagen T., et al (2013) Nicotinic acid adenine dinucleotide phosphate (NAADP)-mediated calcium signaling and arrhythmias in the heart evoked by β-adrenergic stimulation. J. Biol. Chem. 288, 16017–16030 23564460 10.1074/jbc.M112.441246 PMC3668757

[35] Pitt S. J., Funnell T. M., Sitsapesan M., Venturi E., Rietdorf K., Ruas M., Ganesan A., Gosain R., Churchill G. C., Zhu M. X., Parrington J., Galione A., and Sitsapesan R. (2010) TPC2 is a novel NAADP-sensitive Ca^2+^ release channel, operating as a dual sensor of luminal pH and Ca^2+^. J. Biol. Chem. 285, 35039–35046 20720007 10.1074/jbc.M110.156927 PMC2966118

[36] Jha A., Ahuja M., Patel S., Brailoiu E., and Muallem S. (2014) Convergent regulation of the lysosomal two-pore channel-2 by Mg^2+^, NAADP, PI(3,5)P(2) and multiple protein kinases. EMBO J. 33, 501–511 24502975 10.1002/embj.201387035 PMC3989630

[37] Morgan A. J., and Galione A. (2014) Two-pore channels (TPCs): current controversies. BioEssays 36, 173–183 24277557 10.1002/bies.201300118

[38] Jentsch T. J., Hoegg-Beiler M. B., and Vogt J. (2015) Departure gate of acidic Ca^2+^ confirmed. EMBO J. 34, 1737–1739 26022292 10.15252/embj.201591884 PMC4516426

[39] Morris J. K. (1965) A formaldehyde-glutaraldehyde fixative of high osmolality for use in electron microscopy. J. Cell Biol. 27, 1A–149A5857256

[40] Parkesh R., Vasudevan S. R., Berry A., Galione A., Dowden J., and Churchill G. C. (2007) Chemo-enzymatic synthesis and biological evaluation of photolabile nicotinic acid adenine dinucleotide phosphate (NAADP^+^). Org. Biomol. Chem. 5, 441–443 17252124 10.1039/b617344f PMC2518626

[41] Parkesh R., Lewis A. M., Aley P. K., Arredouani A., Rossi S., Tavares R., Vasudevan S. R., Rosen D., Galione A., Dowden J., and Churchill G. C. (2008) Cell-permeant NAADP: a novel chemical tool enabling the study of Ca^2+^ signalling in intact cells. Cell Calcium 43, 531–538 17935780 10.1016/j.ceca.2007.08.006

[42] Liu W., Zi M., Naumann R., Ulm S., Jin J., Taglieri D. M., Prehar S., Gui J., Tsui H., Xiao R. P., Neyses L., Solaro R. J., Ke Y., Cartwright E. J., Lei M., and Wang X. (2011) Pak1 as a novel therapeutic target for antihypertrophic treatment in the heart. Circulation 124, 2702–2715 22082674 10.1161/CIRCULATIONAHA.111.048785 PMC3242076

[43] Naylor E., Arredouani A., Vasudevan S. R., Lewis A. M., Parkesh R., Mizote A., Rosen D., Thomas J. M., Izumi M., Ganesan A., Galione A., and Churchill G. C. (2009) Identification of a chemical probe for NAADP by virtual screening. Nat. Chem. Biol. 5, 220–226 19234453 10.1038/nchembio.150 PMC2659327

[44] Mattiazzi A., and Kranias E. G. (2011) CaMKII regulation of phospholamban and SR Ca^2+^ load. Heart Rhythm 8, 784–787 21111063 10.1016/j.hrthm.2010.11.035 PMC3081991

[45] Aley P. K., Mikolajczyk A. M., Munz B., Churchill G. C., Galione A., and Berger F. (2010) Nicotinic acid adenine dinucleotide phosphate regulates skeletal muscle differentiation via action at two-pore channels. Proc. Natl. Acad. Sci. U.S.A. 107, 19927–19932 21041635 10.1073/pnas.1007381107 PMC2993425

[46] Lu Y., Hao B. X., Graeff R., Wong C. W., Wu W. T., and Yue J. (2013) Two pore channel 2 (TPC2) inhibits autophagosomal-lysosomal fusion by alkalinizing lysosomal pH. J. Biol. Chem. 288, 24247–24263 23836916 10.1074/jbc.M113.484253 PMC3745369

[47] Lear P. V., González-Touceda D., Porteiro Couto B., Viaño P., Guymer V., Remzova E., Tunn R., Chalasani A., García-Caballero T., Hargreaves I. P., Tynan P. W., Christian H. C., Nogueiras R., Parrington J., and Diéguez C. (2015) Absence of intracellular ion channels TPC1 and TPC2 leads to mature-onset obesity in male mice, due to impaired lipid availability for thermogenesis in brown adipose tissue. Endocrinology 156, 975–986 25545384 10.1210/en.2014-1766 PMC4330317

[48] Davis L. C., Morgan A. J., Chen J. L., Snead C. M., Bloor-Young D., Shenderov E., Stanton-Humphreys M. N., Conway S. J., Churchill G. C., Parrington J., Cerundolo V., and Galione A. (2012) NAADP activates two-pore channels on T cell cytolytic granules to stimulate exocytosis and killing. Curr. Biol. 22, 2331–2337 23177477 10.1016/j.cub.2012.10.035 PMC3525857

[49] Hockey L. N., Kilpatrick B. S., Eden E. R., Lin-Moshier Y., Brailoiu G. C., Brailoiu E., Futter C. E., Schapira A. H., Marchant J. S., and Patel S. (2015) Dysregulation of lysosomal morphology by pathogenic LRRK2 is corrected by TPC2 inhibition. J. Cell Sci. 128, 232–238 25416817 10.1242/jcs.164152 PMC4294771

[50] Manford A. G., Stefan C. J., Yuan H. L., Macgurn J. A., and Emr S. D. (2012) ER-to-plasma membrane tethering proteins regulate cell signaling and ER morphology. Dev. Cell 23, 1129–1140 23237950 10.1016/j.devcel.2012.11.004

[51] De Vos K. J., Mórotz G. M., Stoica R., Tudor E. L., Lau K. F., Ackerley S., Warley A., Shaw C. E., and Miller C. C. (2012) VAPB interacts with the mitochondrial protein PTPIP51 to regulate calcium homeostasis. Hum. Mol. Genet. 21, 1299–1311 22131369 10.1093/hmg/ddr559 PMC3284118

[52] Fameli N., Ogunbayo O. A., van Breemen C., and Evans A. M. (2014) Cytoplasmic nanojunctions between lysosomes and sarcoplasmic reticulum are required for specific calcium signaling. F1000Res. 3, 93 25126414 10.12688/f1000research.3720.1 PMC4126599

[53] Grimm M., and Brown J. H. (2010) β-Adrenergic receptor signaling in the heart: role of CaMKII. J. Mol. Cell. Cardiol. 48, 322–330 19883653 10.1016/j.yjmcc.2009.10.016 PMC2896283

[54] Anderson M. E., Brown J. H., and Bers D. M. (2011) CaMKII in myocardial hypertrophy and heart failure. J. Mol. Cell. Cardiol. 51, 468–473 21276796 10.1016/j.yjmcc.2011.01.012 PMC3158288

[55] Khoo M. S., Li J., Singh M. V., Yang Y., Kannankeril P., Wu Y., Grueter C. E., Guan X., Oddis C. V., Zhang R., Mendes L., Ni G., Madu E. C., Yang J., Bass M., et al (2006) Death, cardiac dysfunction, and arrhythmias are increased by calmodulin kinase II in calcineurin cardiomyopathy. Circulation 114, 1352–1359 16982937 10.1161/CIRCULATIONAHA.106.644583

[56] Huke S., Desantiago J., Kaetzel M. A., Mishra S., Brown J. H., Dedman J. R., and Bers D. M. (2011) SR-targeted CaMKII inhibition improves SR Ca^2+^ handling, but accelerates cardiac remodeling in mice overexpressing CaMKIIδC. J. Mol. Cell. Cardiol. 50, 230–238 20971119 10.1016/j.yjmcc.2010.10.014 PMC3018844

[57] Mishra S., Gray C. B., Miyamoto S., Bers D. M., and Brown J. H. (2011) Location matters: clarifying the concept of nuclear and cytosolic CaMKII subtypes. Circ. Res. 109, 1354–1362 21998325 10.1161/CIRCRESAHA.111.248401 PMC3241507

[58] Anderson M. E., Braun A. P., Wu Y., Lu T., Wu Y., Schulman H., and Sung R. J. (1998) KN-93, an inhibitor of multifunctional Ca^++^/calmodulin-dependent protein kinase, decreases early after depolarizations in rabbit heart. J. Pharmacol. Exp. Ther. 287, 996–1006 9864285

[59] Rakovic S., and Terrar D. A. (2002) in Cyclic ADP-Ribose and NAADP (Lee H. C., ed) pp. 319–341, Springer Science and Business Media, New York

[60] Kannt A., Sicka K., Kroll K., Kadereit D., and Gögelein H. (2012) Selective inhibitors of cardiac ADPR cyclase as novel anti-arrhythmic compounds. Naunyn Schmiedebergs Arch. Pharmacol. 385, 717–727 22526470 10.1007/s00210-012-0750-2 PMC3367138

[61] Higashida H., Egorova A., Higashida C., Zhong Z. G., Yokoyama S., Noda M., and Zhang J. S. (1999) Sympathetic potentiation of cyclic ADP-ribose formation in rat cardiac myocytes. J. Biol. Chem. 274, 33348–33354 10559213 10.1074/jbc.274.47.33348

[62] Xie G. H., Rah S. Y., Kim S. J., Nam T. S., Ha K. C., Chae S. W., Im M. J., and Kim U. H. (2005) ADP-ribosyl cyclase couples to cyclic AMP signaling in the cardiomyocytes. Biochem. Biophys. Res. Commun. 330, 1290–1298 15823583 10.1016/j.bbrc.2005.03.114

[63] Luo W., Wolska B. M., Grupp I. L., Harrer J. M., Haghighi K., Ferguson D. G., Slack J. P., Grupp G., Doetschman T., Solaro R. J., and Kranias E. G. (1996) Phospholamban gene dosage effects in the mammalian heart. Circ. Res. 78, 839–847 8620604 10.1161/01.res.78.5.839

